# Impaired lung function is associated with non-alcoholic fatty liver disease independently of metabolic syndrome features in middle-aged and elderly Chinese

**DOI:** 10.1186/s12902-017-0168-4

**Published:** 2017-03-22

**Authors:** Li Qin, Weiwei Zhang, Zhen Yang, Yixin Niu, Xiaoyong Li, Shuai Lu, Yin Xing, Ning Lin, Hongmei Zhang, Guang Ning, Jiangao Fan, Qing Su

**Affiliations:** 1 Department of Endocrinology, Xinhua Hospital Chongming Branch, Shanghai Jiao Tong University School of Medicine, Shanghai, China; 2 Department of Endocrinology, Xinhua Hospital, Shanghai Jiao Tong University School of Medicine, 1665 Kongjiang Road, Shanghai, China; 3 Department of Endocrinology and Metabolism, Key Laboratory for Endocrine and Metabolic Diseases of Ministry of Health, RuiJin Hospital, Shanghai Jiao Tong University School of Medicine, E-Institute of Shanghai Universities, Shanghai, China; 4 Department of Gastroenterology, Shanghai Key Laboratory of Children’s Digestion and Nutrition, Xinhua Hospital, Shanghai Jiaotong University School of Medicine, Shanghai, China

**Keywords:** Lung function, Non-alcoholic fatty liver disease, Chinese, Metabolic risk factors

## Abstract

**Background:**

Associations between lung function and non-alcoholic fatty liver disease (NAFLD) have been reported. However, evidence from large-scale populations about the relationship is scarce. The objective of the study was to evaluate the relationship between lung function and NAFLD in middle-aged and elderly Chinese.

**Methods:**

A total of 1842 participants aged 40 years or older were recruited from Chongming District, Shanghai, China. Lung function, evaluated by forced vital capacity (FVC) and forced expiratory volume in one second (FEV1) was measured with standard spirometry. The NAFLD was evaluated by ultrasonography.

**Results:**

The subjects with NAFLD had lower FVC (% predicted) (0.85 ± 0.26 vs. 0.90 ± 0.28, *p* < 0.001) and FEV1 (% predicted) (0.93 ± 0.29 vs. 0.98 ± 0.34, *p* < 0.001) than non-NAFLD. After adjusting for potential risk factors, the lowest quartile of FVC (% predicted) and FEV1 (% predicted) was associated with increased prevalence of NAFLD, with the fully adjusted odds ratio of 1.37 and 1.24 (95% confidence interval [CI] 1.18–1.97, *p* < 0.001, 95% CI 1.11–1.87, *p* = 0.009), respectively.

**Conclusions:**

Impaired lung function is associated with non-alcoholic fatty liver disease, independent of conventional metabolic risk factors.

## Background

Nonalcoholic fatty liver disease (NAFLD) is characterized by excessive hepatic fat accumulation of patients who have no history of alcohol abuse [1]. Recently, the combination of overnutrition condition and less physical activity have made NAFLD become the most common disease of chronic liver damage, with increased prevalence of obesity, diabetes, and metabolic syndrome in developed and developing counties [2]. The traditional risk factors of NAFLD, such as central obesity, insulin resistance, systemic inflammation, current smoking, diabetes, and oxidative stress, contribute to, but cannot fully explain the increased risk of NAFLD in the general population [3–5].

**Fig. 1:**
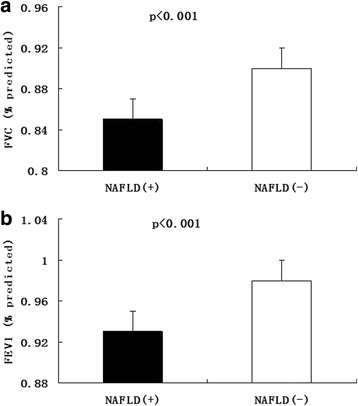
The levels of FVC (% pred) and FEV1 (% pred) in subjects with NAFLD and without NAFLD. Data are shown as means ± SE after adjustment for age and sex. (A for FVC and B for FEV1)

Recently, lung function parameters, estimated by forced vital capacity (FVC) and forced expiratory volume in one second (FEV1) were proved to be well associated with the development of diabetes, cardiovascular disease, inflammation process and metabolic syndrome [6–25]. NAFLD has been considered as a hepatic manifestation of the metabolic syndrome and is associated with various metabolic abnormalities, including hyperlipidemia, central obesity, and type 2 diabetes [1, 26, 27]. So, reduced lung function may link to an increased risk of NAFLD. In a previous study, association of reduced lung function with NAFLD was detected among men in a health examination program [28]. However, evidence from large-scale populations about the relationship between reduced lung function and NAFLD is scarce. In addition, it is unclear whether the association can be observed in Chinese population.

For this purpose, the aims of this study were to test the hypotheses that reduced lung function is independently associated with NAFLD in a cross-sectional population study of 1,842 middle-aged and older Chinese subjects.

## Methods

### Study population

In 2011, China launched a national survey of Risk Evaluation of cAncers in Chinese diabeTic Individuals: a lONgitudinal (REACTION) study, which was conducted among 259,657 adults, aged 40 years and older in 25 communities across mainland China, from 2011 to 2012 [29]. The data presented in this article are based on the baseline survey of subsamples from Shanghai in eastern China [30, 31]. All studied individuals came from the Chongming District in Shanghai, China. There were 9930 participants who had complete information about age; sex; smoking and alcohol consumption habits; and a medical history including the use of medications, BMI, and a hepatic ultrasonic examination. Participants meeting the following criteria were excluded: 1) those with a history of known liver diseases such as hepatitis, cirrhosis, or malignancy; 2) those with alcohol consumption greater than 140 g/wk for men and 70 g/wk for women. Thus, a total of 8850 participants were eventually included in this analysis. Of these, two communities participants received lung function test. 1,842 participants were eventually included in the analysis. The protocol was approved by the Institutional Review Board of Xinhua Hospital affiliated with Shanghai Jiao-Tong University School of Medicine.

### Data collection

A standardized questionnaire was used by trained physicians to collect information such as age; sex; current smoking (yes/no); current drinker (yes/no). Physical activity level was classified as low, moderate, or high according to the International Physical Activity Questionnaire scoring protocol. According to participants’ responses to the corresponding questions, family history of diabetes was classified as yes or no.

The details of anthropometric measurements including height, weight, waist circumference, hip circumference were carried by trained physicians. Blood pressure was measured at the right arm with an automated electronic device (OMRON Model1 Plus; Omron Company, Kyoto, Japan) three times consecutively with 1 min intervals after at least 5 min rest in the seated position; the three readings were averaged for analysis. Body mass index (BMI) was calculated as weight in kilograms divided by the square of height in meters.

All subjects were assessed after overnight fasting for at least 10 h, Overnight fasting and 2 h OGTT (Oral Glucose Tolerance Test) 75 g glucose blood samples were collected in tubes containing EDTA and were centrifuged at 4 °C and stored at−80 °C until analysis. The fasting glucose, glucose 2 h after oral glucose tolerance test, total cholesterol (TC), triglycerides, low-density lipoprotein (LDL) cholesterol and high-density lipoprotein (HDL) cholesterol were measured on an automatic analyzer (Hitachi 7080; Tokyo, Japan). Fasting insulin was determined by RIA (Linco Research, St. Charles, MO). The homeostasis model assessment of insulin resistance (HOMA-IR) was calculated according to the equation described by Matthews et al. [32].

### Definition of NAFLD

Hepatic ultrasonic examination was performed on all participants by two trained ultrasonographists who were blinded to the clinical and laboratory data, using a high-resolution B-mode tomographic ultrasound system (Esaote Biomedica SpA, Italy) with a 3.5-MHz probe. Diagnosis of fatty liver by ultrasonography was defined by the presence of at least two of three abnormal findings: diffusely increased echogenicity of the liver relative to the kidney, ultrasound beam attenuation, and poor visualization of intrahepatic structures. NAFLD was diagnosed by hepatic ultrasound after the exclusion of alcohol abuse and other liver diseases.

### Lung function measurements

Lung function tests including FVC and FEV1 were conducted by a trained physician using Electronic Spirometer (Model BF-II, Jintan, China). Each participant received at least two tests (with acceptable maneuvers) at a seated position and with nose clips in place. The predicted values for FVC and FEV1 were calculated from the following equations obtained in a representative sample of Chinese population [25].

Predicted FVC of man = −4.33058–(0.01326× age [years]) + (0.04669× height [cm]) + (0.01664× weight [kg]).

Predicted FVC of woman = −4.79287– (0.01326× age [years]) + (0.04669× height [cm]) + (0.01664× weight [kg]).

Predicted FEV1 of man = −3.65523– (0.01850× age [years]) + (0.04283× height [cm]) + (0.009228832× weight [kg]).

Predicted FEV1 of woman = −4.04947– (0.01850× age [years]) + (0.04283× height [cm]) + (0.009228832× weight [kg]).

The percentage of predicted values for FEV1, FEV1 (% pred), equals to FEV1 devided by the predicted values of FEV1. The percentage of predicted values for FVC, FVC (% pred), equals to FVC divided by the predicted values of FVC. The ratio of FEV1 to FVC was calculated.

### Statistical analysis

Normally distributed data were expressed as means ± SD, whereas variables with a skewed distribution were reported as median (interquartile range) and log transformed to approximate normality before analysis. Comparisons of means and proportions were performed with the standard normal z and *χ*2 tests, respectively. Multivariate logistic regression models were used to estimate the odds ratios (ORs) for NAFLD. Potential confounding variables including age, gender, current smoking, family history of diabetes, systolic blood pressure, diastolic blood pressure, fasting plasma glucose, 2 h OGTT plasma glucose, hemoglobin A1c, waist circumference, BMI, HOMA-IR, TG, TC, LDL-c and HDL-c were controlled in the regression models. All statistical analysis were performed with the SPSS Statistical Package (version 13.0; SPSS Inc., Chicago, IL). *P* < 0.05 was considered statistically significant.

## Results

### Characteristics of participants according to FVC (% pred) or FEV1 (% pred) quartile

Mean values of FVC (% pred), FEV1 (% pred) in subjects with NAFLD were significantly lower than in those without (0.85 ± 0.26 vs. 0.90 ± 0.28, 0.93 ± 0.29 vs. 0.98 ± 0.34; both *p* < 0.001) (Fig. 1). Individuals with NAFLD were elder, more likely to be metabolic syndrome, current drinker, and heavy smoking, and had higher levels of BMI, SBP, DBP, waist circumference, hip circumference, waist-hip ratio, fasting plasma glucose FPG, postprandial 2-h plasma glucose, A1C, HOMA-IR, TG, TC, LDL-c, AST, ALT and GGT (all *p* values < 0.001), and had lower levels of HDL-c (*p* < 0.001) (Table 1).

**Table 1:** Baseline characteristics of the study participants, grouped according to NAFLD status

	Without NAFLD (*n* = 1164)	With NAFLD (*n* = 678)	*P* value
ATPIII-defined metabolic syndrome n(%)	433(37.21)	526(77.64)	<0.001
Sex n (% men)	376(32.3)	213(31.4)	0.38
Age (years)^b^	55.38 ± 8.10	56.97 ± 7.55	<0.001
BMI (kg/m2)	23.32 ± 2.95	26.47 ± 6.33	<0.001
Currents smokers n (%)	275(23.63)	178(26.30)	<0.001
SBP (mmHg)	127.49 ± 20.64	133.10 ± 20.98	<0.001
DBP (mmHg)	78.99 ± 10.53	81.77 ± 10.34	<0.001
Waist circumference (cm)	81.18 ± 10.46	89.42 ± 8.70	<0.001
Hip circumference (cm)	94.12 ± 6.06	99.05 ± 6.95	<0.001
Waist-hip ratio	0.86 ± 0.14	0.90 ± 0.07	<0.001
Fasting plasma glucose (mmol/l)	6.02 ± 1.45	6.65 ± 1.93	<0.001
2 h PG (mmol/L)	7.87 ± 3.40	9.17 ± 4.22	<0.001
A1C(%)	5.82 ± 0.86	6.25 ± 1.14	<0.001
HOMA-IR	1.47 (1.13–1.92)	2.36 (1.63–3.27)	<0.001
Triglycerides (mmol/l)	1.38 ± 0.95	2.16 ± 1.54	<0.001
HDL-cholesterol (mmol/l)	1.29 ± 0.33	1.15 ± 0.28	<0.001
LDL-cholesterol (mmol/l)	2.55 ± 0.74	2.70 ± 0.80	<0.001
AST(units/l)	15.92 ± 8.67	22.29 ± 12.54	<0.001
ALT(units/l)	14.15 ± 10.03	21.19 ± 15.87	<0.001
GGT(units/l)	24.09 ± 28.30	38.61 ± 48.45	<0.001

*SBP* systolic blood pressure, *DBP* diastolic blood pressure, *BMI* body mass index; *2hPG* postprandial 2-h plasma glucose, *HbA1C* Glycated hemoglobin, *LDL* Low-density lipoprotein, *HDL* high--density lipoprotein, *ALT* Alanine aminotransferase, *AST* aspartate aminotransferase, GGT γ-glutamyltransferase

^a^Data are presented as mean ± SD, median (interquartile range), or number (percent); *P* value was calculated after adjustment for age, gender

^b^Not adjusted for itself

^c^This variables was log transformed before analysis

When analyzed by quartiles of FVC (% pred) or FEV1 (% pred) levels, as summarized in Tables 2 and 3, the subjects with lower FVC (% pred) or FEV1 (% pred) were more likely to be more metabolic syndrome (*p* < 0.001), more smoker (*p* < 0.001), more drinker (*p* < 0.001), more aged (*p* < 0.001), With respect to metabolic parameters, the subjects in the higher FVC (% pred) or FEV1 (% pred) quartiles exhibited low er levels of LDL cholesterol (*p* < 0.001). However, elevated FVC (% pred) or FEV1 (% pred) levels showed no association with the regular exerciser (*p* > 0.05).

**Table 2:** Characteristic according to quartiles of FVC (% predicted)^a^

	Quartile 1 (*n* = 450)	Quartile 2 (*n* = 471)	Quartile 3 (*n* = 460)	Quartile 4 (*n* = 471)	*P* value
FVC (% predicted)	0.63 ± 0.09	0.77 ± 0.25	0.87 ± 0.03	1.22 ± 0.28	<0.001
FEV1 (% predicted)	0.70 ± 0.18	0.86 ± 0.15	1.01 ± 0.26	1.27 ± 0.31	<0.001
FEV1/FVC ratio	0.77 ± 0.22	0.77 ± 0.22	0.76 ± 0.23	0.74 ± 0.24	<0.001
MS (n, %)	294(65.33)	271(57.53)	253(55.00)	232(49.26)	<0.001
NAFLD (n, %)	193(42.89)	188(39.92)	160(34.78)	137(29.09)	<0.001
Age (years)^b^	56.64 ± 8.23	55.65 ± 7.87	55.37 ± 7.48	56.31 ± 7.91	0.007
Male (n, %)	45(10.00)	78 (16.56)	168(36.52)	384(81.53)	<0.001
Current smoking (n, %)	51(11.33)	66(14.01)	87(18.91)	166(35.24)	<0.001
Current drinker (n, %)	61(13.56)	88(18.68)	86(18.70)	183(38.85)	<0.001
Regular exerciser (n, %)	265(58.89)	282(59.87)	275(59.78)	284(60.29)	0.978
BMI (kg/m^2^)	24.82 ± 3.97	25.09 ± 3.59	24.48 ± 3.27	24.73 ± 3.34	0.102
SBP (mmHg)	137.56 ± 19.42	134.32 ± 18.82	131.51 ± 19.15	135.78 ± 18.95	<0.001
DBP (mmHg)	82.40 ± 10.44	82.16 ± 10.87	80.98 ± 9.98	82.53 ± 10.57	<0.001
WC (cm)	86.54 ± 11.48	85.76 ± 11.11	83.93 ± 10.31	84.73 ± 10.45	0.002
FPG (mmol/L)	6.54 ± 1.87	6.65 ± 2.21	6.32 ± 1.75	6.31 ± 1.36	0.011
2 h PG (mmol/L)	9.42 ± 4.23	9.43 ± 4.57	8.70 ± 4.01	8.38 ± 3.61	<0.001
A1C (%)	5.90 ± 1.10	5.94 ± 1.23	5.75 ± 1.01	5.67 ± 0.83	<0.001
HOMA-IRc	2.32(1.44–3.31)	1.84 (1.30–2.68)	1.75(1.22–2.50)	1.66(1.18–2.32)	0.029
eGFR	124.75 ± 25.25	122.57 ± 21.90	120.30 ± 19.95	114.73 ± 21.85	<0.001
Triglycerides (mmol/L)^c^	1.88 ± 1.27	1.76 ± 1.30	1.63 ± 1.05	1.89 ± 1.65	0.009
TC (mmol/L)	5.14 ± 0.96	5.07 ± 0.88	4.87 ± 0.85	4.86 ± 0.88	<0.001
HDL-c (mmol/L)	1.34 ± 0.32	1.36 ± 0.31	1.31 ± 0.32	1.29 ± 0.33	0.004
LDL-c (mmol/L)	2.84 ± 0.75	2.81 ± 0.74	2.73 ± 0.70	2.71 ± 0.72	<0.001
ALT (units/l)	21.37 ± 17.36	21.08 ± 15.98	19.78 ± 14.06	17.82 ± 11.15	<0.001
AST (units/l)	25.35 ± 13.05	24.28 ± 12.53	24.13 ± 10.75	22.14 ± 7.31	<0.001
GGT (units/l)	42.19 ± 42.29	36.22 ± 51.10	30.96 ± 33.42	29.91 ± 37.36	<0.001

*SBP* systolic blood pressure, *DBP* diastolic blood pressure, *BMI* body mass index, *OGTT* Oral Glucose Tolerance Test, *FPG* Fasting Plasma Glucose, *2 h PG* postprandial 2-h Plasma Glucose, *HbA1C* Glycated hemoglobin, *LDL* Low-density lipoprotein, *HDL* high--density lipoprotein, *ALT* Alanine aminotransferase, *AST* aspartate aminotransferase, *GGT* γ-glutamyltransferase

^a^Data are presented as mean ± SD, median (interquartile range), or number (percent); *P* value was calculated after adjustment for age, gender

^b^Not adjusted for itself

^c^This variables was log transformed before analysis

**Table 3:** Characteristic according to quartiles of FEV1 (% predicted)^a^

	Quartile 1 (*n* = 454)	Quartile 2 (*n* = 467)	Quartile 3 (*n* = 464)	Quartile 4 (*n* = 467)	*P* value
FEV1 (%)	0.66 ± 0.11	0.83 ± 0.03	0.95 ± 0.05	1.42 ± 0.26	<0.001
FVC (%)	0.66 ± 0.14	0.79 ± 0.12	0.92 ± 0.20	1.13 ± 0.30	<0.001
FEV1/FVC ratio	73.21 ± 22.91	77.23 ± 22.01	76.63 ± 22.81	78.02 ± 22.61	<0.001
MS (n, %)	282(62.11)	273(58.46)	255(54.96)	240(51.39)	<0.001
NAFLD (n, %)	200(44.05)	171(36.62)	162(34.91)	145(30.79)	<0.001
Age (years)^b^	56.37 ± 8.22	55.51 ± 7.72	55.17 ± 8.12	56.68 ± 7.50	0.004
Male (n, %)	47(10.35)	82(17.56)	164(35.34)	382(81.80)	<0.001
Current smoking (n, %)	55(12.11)	68(14.56)	87(18.75)	159(34.05)	<0.001
Current drinker (n, %)	64(14.10)	90(19.27)	89(19.18)	175(37.47)	<0.001
Regular exerciser (n, %)	263(57.93)	285(61.03)	272(58.62)	287(61.46)	0.872
BMI (kg/m^2^)	24.81 ± 3.93	24.76 ± 3.64	25.05 ± 3.42	24.74 ± 3.32	0.556
SBP (mmHg)	136.28 ± 19.26	135.84 ± 19.25	134.38 ± 18.12	133.54 ± 20.55	0.002
DBP (mmHg)	82.50 ± 10.43	82.12 ± 10.49	82.06 ± 10.36	81.90 ± 10.81	<0.001
WC (cm)	86.04 ± 11.68	85.06 ± 11.34	86.05 ± 10.27	84.70 ± 10.29	0.015
FPG (mmol/L)	6.55 ± 1.88	6.50 ± 3.64	6.48 ± 1.90	6.36 ± 1.49	0.004
2 h PG (mmol/L)	9.30 ± 4.20	9.10 ± 4.39	9.03 ± 4.09	8.47 ± 3.79	0.017
A1C(%)	5.88 ± 1.07	5.88 ± 1.14	5.83 ± 1.11	5.70 ± 0.93	0.035
HOMA-IR^c^	2.30(1.42–3.33)	1.79(1.28–2.63)	1.70(1.23–2.52)	1.64(1.15–2.31)	0.025
eGFR	124.85 ± 25.05	121.73 ± 21.24	119.85 ± 21.90	115.14 ± 21.27	<0.001
Triglycerides (mmol/L)	1.86 ± 1.26	1.73 ± 1.35	1.80 ± 1.38	1.77 ± 1.42	0.035
TC (mmol/L)	5.06 ± 0.93	4.95 ± 1.02	4.98 ± 0.84	4.92 ± 0.82	0.125
HDL–c (mmol/L)	1.33 ± 0.32	1.32 ± 0.30	1.32 ± 0.31	1.32 ± 0.31	0.031
LDL-c (mmol/L)	2.83 ± 0.75	2.79 ± 0.79	2.70 ± 0.67	2.69 ± 0.68	0.007
ALT(units/l)	21.22 ± 14.07	21.15 ± 14.28	19.94 ± 13.53	18.17 ± 13.09	<0.001
AST(units/l)	24.97 ± 12.85	24.31 ± 12.66	24.03 ± 11.49	22.47 ± 10.29	<0.001
GGT(units/l)	41.25 ± 37.73	38.07 ± 43.25	31.24 ± 34.71	28.99 ± 36.52	<0.001

*SBP* systolic blood pressure, *DBP* diastolic blood pressure, *BMI* body mass index, *OGTT* Oral Glucose Tolerance Test, *FPG* Fasting Plasma Glucose, *2 h PG* postprandial 2-h Plasma Glucose, *HbA1C HbA1C* hemoglobin A1C, *eGFR* estimate the glomerular filtration rate, *LDL* Low-density lipoprotein, *HDL* high--density lipoprotein, *ALT* Alanine aminotransferase, *AST* aspartate aminotransferase, *GGT* γ-glutamyltransferase

^a^Data are presented as mean ± SD, median (interquartile range), or number (percent); *P* value was calculated after adjustment for age, gender

^b^Not adjusted for itself

^c^This variables was log transformed before analysis

### Association between quartiles of FVC (% pred) and FEV1 (% pred) and NAFLD

As shown in Table 4, the lowest quartile of FVC (% pred) and FEV1 (% pred) was associated with increased odds of NAFLD, with age- and sex-adjusted odds ratio (OR) of 1.82 and 1.74, respectively (95% confidential interval (CI), 1.38–2.39 and 95% CI, 1.32–2.28; both *p* < 0.001). Further adjustments for current smoking, family history of diabetes systolic blood pressure, diastolic blood pressure, fasting plasma glucose, 2 h OGTT plasma glucose, Hemoglobin A1c, waist circumference, BMI, HOMA-IR, TG, TC, LDL-c and HDL-c did not eliminate the associations (OR, 1.37, 95% CI, 1.18–1.97, *p* < 0.001 and OR,1.24, 95% CI, 1.11–1.87, *p* = 0.009).

**Table 4:** Odds ratio for the non-alcoholic fatty liver disease according to quartiles of FVC (% pred) or FEV1 (% pred)

	FVC(% pred)				
	Quartile 1	Quartile 2	Quartile 3	Quartile 4	*P* value
Model 1	1.82(1.38–2.39)	1.60(1.23–2.12)	1.29(0.97–1.70)	1	<0.001
Model 2	1.65(1.27–2.24)	1.39(1.18–2.01)	1.12(0.87–1.52)	1	<0.001
Model 3	1.37(1.18–1.97)	1.19(1.08–1.82)	1.04(0.79–1.28)	1	<0.001
	FEV1(% pred)				
	Quartile 1	Quartile 2	Quartile 3	Quartile 4	*P* value
Model 1	1.74(1.32–2.28)	1.27(0.96–1.67)	1.18(0.90–1.55)	1	<0.001
Model 2	1.48(1.24–2.12)	1.13(0.89–1.48)	1.09(0.81–1.39)	1	0.004
Model 3	1.24(1.11–1.87)	1.07(0.76–1.21)	1.03(0.72–1.19)	1	0.009

Model 1, adjusted for age and sex; model 2, further adjusted for current smoking, family history of diabetes systolic blood pressure and diastolic blood pressure; model 3, further adjusted for fasting plasma glucose, 2 h OGTT plasma glucose, Hemoglobin A1c, waist circumference, BMI, HOMA-IR, TG, TC, LDL-c and HDL-c

## Discussion

In the present study, we found that impaired lung function as measured by FVC and FEV1 was significantly and inversely associated with prevalence of NAFLD in a middle-aged and elderly population without chronic pulmonary diseases after adjustment for a wide range of variables including age, gender, current smoking, family history of diabetes, systolic blood pressure, diastolic blood pressure, fasting plasma glucose, 2 h OGTT plasma glucose, hemoglobin A1c, waist circumference, BMI, HOMA-IR and lipid profile.

Our results are consistent with previous study that restrictive lung function (reduced FVC and FEV1) but not obstructive pulmonary function (FEV1-to-FVC ratio) is associated with the development of NAFLD [28]. The underlying mechanisms relating reduced lung function to this type of metabolic disorder remain unclear; integration of inflammatory process and metabolic pathways in NAFLD patients may be a pivotal underlying mechanism link between reduced pulmonary function and incident NAFLD. Previous studies have demonstrated that a strong association between both restrictive and obstructive lung patterns and inflammatory markers [33, 34]. As we best known, low-grade systemic inflammation play a causal role in the development of NAFLD. Thus, the inflammatory process may contribute to the association between reduced lung function and NAFLD. However, the measurement of inflammatory markers was absent, limiting the ability to access the role of this factor in the association in the present study.

In our study, we observed that the positive association of FVC (% pred) in particular and FEV1 (% pred) with metabolic abnormalities and components of the insulin resistance syndrome, which is consistent with several previous studies that have reported associations between restrictive lung patterns with glucose metabolism and metabolic syndrome [6–23]. Moreover, our study also demonstrated that FVC (% pred) and FEV1 (% pred) were associated with insulin sensitivity as measured by the HOMA-IR. It has been well demonstrated that insulin resistance plays a key role in the development of NAFLD [35, 36]. Metabolic risk factors closely associated with insulin resistance (BMI, glucose, waist circumference, blood pressure, triglycerides, and HDL cholesterol) may affect the association of FVC (% pred), FEV1 (% pred) and NAFLD. However, our further analysis indicated that the effects of reduced lung function on NAFLD were independent of metabolic syndrome features.

The strengths of this study include the community-based sample, standardized spirometric techniques, extensive data on potential confounders, and a large sample size that increased precision and permitted multiple statistical adjustments. However, several limitations of our study have to be addressed. First, due to the cross-sectional nature of the current study, no causal inference can be drawn. Prospective studies are needed to clarify their precise interrelationship. Also, it has yet to be seen whether our results in middle-aged and older Chinese subjects can be generalized to younger populations or other ethnic groups. Secondly, liver biopsies, the best diagnostic tool for confirming NAFLD, were not available in our participants. The diagnosis of NAFLD was based on ultrasonic examination, which is not sensitive enough to detect mild steatosis. However, this method is the most widely used noninvasive technique to detect fatty infiltration of the liver in clinical practice and epidemiological studies, and it has been reported to have a sensitivity of 89% and specificity of 93% for the identification of fatty liver [37]. Third, the lack of inflammatory markers, which precluded more detailed investigations of the causal pathway. Furthermore, sleep duration/quality and symptoms of sleep apnea which was related to both insulin resistance and lung function may affect the association of impaired lung function and NAFLD, however, due to study design defect, we couldn’t further analyze the effect in this study.

## Conclusions

We have found that impaired lung function was associated with NAFLD in middle-aged and elderly Chinese population. These findings suggest the need to screen impaired of lung function in people without respiratory disease for the presence of NAFLD.

## Abbreviations

ALT: Alanine aminotransferase
AST: Aspartate aminotransferase
BMI: Body mass index
FEV1: Forced expiratory volume in one second
FVC: Forced vital capacity
GGT: γ-glutamyltransferase
HDL: High-density lipoprotein cholesterol
HOMA-IR: Homeostasis model assessment of insulin resistance
LDL: Low-density lipoprotein cholesterol
NAFLD: Nonalcoholic fatty liver disease
Ors: Odds ratios.
TC: Total cholesterol
